# Targeted Mutation of the Mouse *Grp94* Gene Disrupts Development and Perturbs Endoplasmic Reticulum Stress Signaling

**DOI:** 10.1371/journal.pone.0010852

**Published:** 2010-05-26

**Authors:** Changhui Mao, Miao Wang, Biquan Luo, Shiuan Wey, Dezheng Dong, Robin Wesselschmidt, Stephen Rawlings, Amy S. Lee

**Affiliations:** 1 Department of Biochemistry and Molecular Biology, University of Southern California Norris Comprehensive Cancer Center, University of Southern California Keck School of Medicine, Los Angeles, California, United States of America; 2 Eli and Edythe Broad Center for Regenerative Medicine and Stem Cell Research, University of Southern California Keck School of Medicine, Los Angeles, California, United States of America; Technical University Munich, Germany

## Abstract

Glucose-regulated protein 94 (GRP94) is one of the most abundant endoplasmic reticulum (ER) resident proteins and is the ER counterpart of the cytoplasmic heat shock protein 90 (HSP90). GRP94, a component of the GRP78 chaperone system in protein processing, has pro-survival properties with implicated function in cancer progression and autoimmune disease. Previous studies on the loss of GRP94 function showed that it is required for embryonic development, regulation of toll-like receptors and innate immunity of macrophages. Here we report the creation of mouse models targeting exon 2 of the *Grp94* allele that allows both traditional and conditional knockout (KO) of *Grp94*. In this study, we utilized the viable *Grp94*+/+ and +/− mice, as well as primary mouse embryonic fibroblasts generated from them as experimental tools to study its role in ER chaperone balance and ER stress signaling. Our studies reveal that while *Grp94* heterozygosity reduces GRP94 level it does not alter ER chaperone levels or the ER stress response. To study the effect of complete loss of GRP94 function, since homozygous GRP94 KO leads to embryonic lethality, we generated *Grp94*−/− embryonic stem cells. In contrast to *Grp94* heterozygosity, complete knockout of GRP94 leads to compensatory upregulation of the ER chaperones GRP78, calnexin and calreticulin but not protein disulphide isomerase. Unexpectedly, loss of GRP94 leads to significant decrease in the level of ER-stress induced spliced form of XBP-1 protein, a downstream target of the IRE1 signaling pathway. Furthermore, from analysis of microarray database and immunohistochemical staining, we present predictions where GRP94 may play an important role in specific adult organ homeostasis and function.

## Introduction

Glucose-regulated protein 94 (GRP94), also referred to as endoplasmin, CaBP4, ERp99 and gp96, is the most abundant endoplasmic reticulum (ER) resident glycoprotein accounting for 5 to 10% of the ER content [1]–[6]. Interestingly, while GRP94 is highly conserved in all vertebrates, some invertebrates such as *C. elegans*, *Drosophila* and in plants, it has not been found in yeast [2], [7], [8]. Recently, a GRP94-like protein was found in *E. coli* as the receptor of ompA protein [9]. GRP94 is the ER counterpart of heat shock protein 90 (HSP90) and shares about 50% amino acid homology and similar structural organization. GRP94 exists as homodimers containing an N-terminal ATP-binding domain, peptide-binding domain and C-terminal dimerization domain [2], [10]–[12]. The N-terminus of GRP94 also contains a peptide binding site [13] and ATP binding is reported to dictate the conformation of the N-terminus and regulate its ability to form quaternary structural interactions [14], [15]. Recent mutational analysis further established that the ATPase activity of GRP94 is essential for chaperone activity [16].

GRP94 is coordinately regulated with GRP78, a major ER chaperone that also acts as a master regulator of ER stress signaling [17]–[19]. GRP94 and GRP78 are components of an ER chaperone system [20], [21]; they share common promoter regulatory sequences and are inducible by a variety of ER stress conditions at the transcriptional level [22]–[24]. Similar to GRP78, GRP94 has been implicated as an anti-apoptotic protein conferring survival advantage to cells subjected to ER or chemotoxic stress [1], [25], [26]. However, unlike GRP78 which associates with nascent polypeptides and early folding intermediates, GRP94 binds substrates after they have been released from GRP78 [27] and appears to have a more limited number protein clients including some toll-like receptors and integrins [27]–[29].

During mouse embryonic development both GRP94 and GRP78 transcripts are expressed in very early embryos [30] and both proteins are detected at high levels at later stages during organogenesis [31], [32]. Interestingly, heterozygous knockout of *Grp78* triggers upregulation of GRP94 and other ER chaperones but not activation of other unfolded protein response (UPR) targets such as CHOP and XBP-1 splicing. Although *Grp78*+/− heterozygotes are viable and exhibit no phenotypical abnormality, homozygous knockout of *Grp78* leads to lethality at embryonic day 3.5 (E3.5) [33]. Null mutation of *Grp94* in mice also results in embryonic lethality, but later in development around E7 [28], [34]. *Grp94*−/− embryonic stem cells (ESCs) are hypersensitive to serum deprivation and not able to differentiate into muscle cells due to deficiency in secretion of insulin-like growth factor (IGF) II [34], [35]. Despite these advances, the *in vivo* function of GRP94, which is not only a major ER chaperone but also an ER calcium binding protein, is just emerging. Here we report the creation of mouse models targeting exon 2 of the *Grp94* allele that allow both traditional and conditional knockout (KO) of GRP94. We examined the requirement of GRP94 in mouse embryonic development and the experimental conditions required for establishment of *Grp94−/−* mouse ESCs. Using primary mouse embryonic fibroblasts (MEFs) and ESCs derived from the *Grp94*+/+, +/− and −/− embryos, we discovered that in response to ER stress, GRP94 depletion results in compensatory upregulation of specific ER chaperones and suppression of ER stress-induced splicing of XBP-1, a downstream target of IRE1 signaling pathway. Furthermore, from analysis of microarray database and immunohistochemical staining of GRP94 expression in mouse adult organs, we present predictions where GRP94 may play an important role in specific adult organ homeostasis and function.

## Results

### Creation of *Grp94* Floxed and Knockout Alleles for Targeted Mutation of GRP94

To elucidate the *in vivo* function of GRP94, we created targeted mutation of *Grp94* taking advantage of the cre-loxP and the flp-FRT systems, which allows both traditional as well as conditional knockout of the targeted allele [36], [37]. In our mouse models, inactivation of the *Grp94* gene was achieved through deletion of exon 2, which contains 103 base pairs (bp). Since exon 1 only has 49 bp and the deletion of exon 2 will cause an early frameshift mutation, at most a truncated peptide of 26 amino acids is generated if the mRNA escapes the premature termination codon mediated mRNA degradation system [38]. This abolishes the expression of GRP94 without generating any residual functional domain.

**Figure 1:**
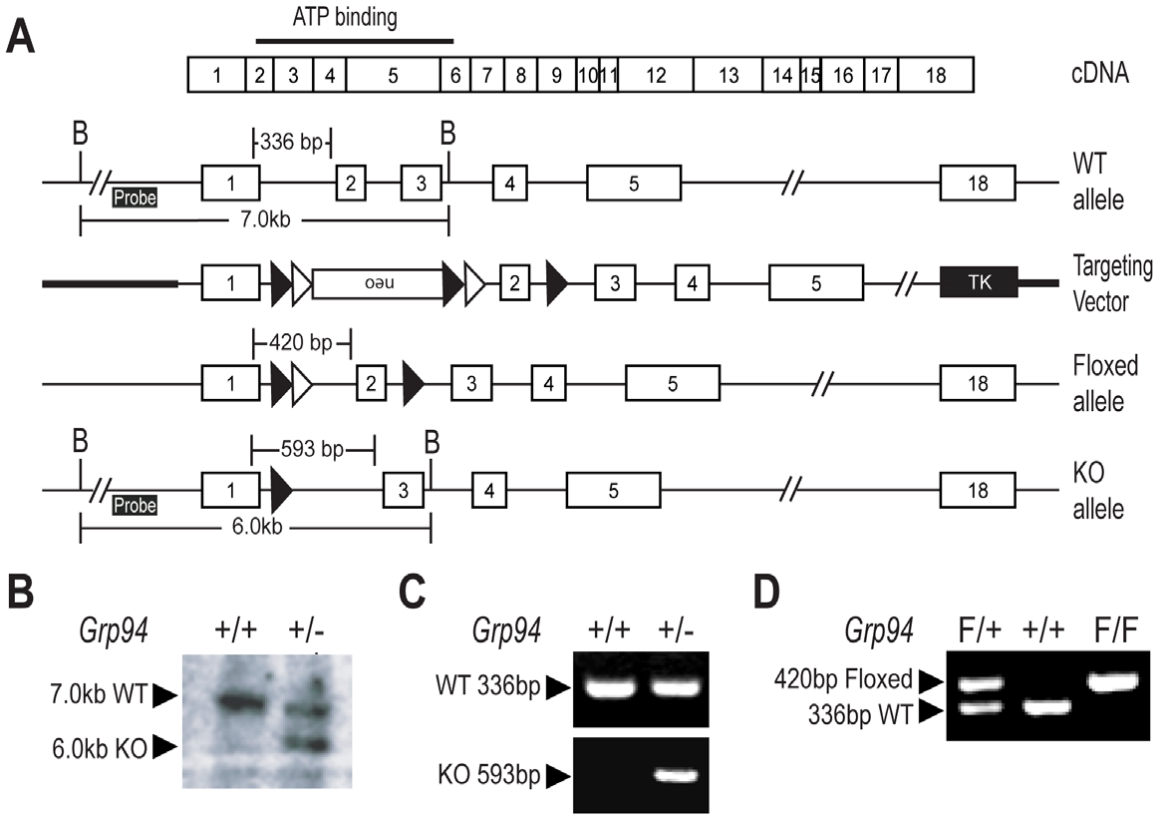
Creation of *Grp94* knockout (KO) and floxed (F/F) mice. (A) Schematic drawings of the *Grp94* exon map, the wild-type (WT) (+) allele, the loxP-FRT targeting vector, the Floxed (F) allele and the KO (−) allele. The ATP-binding domain of GRP94, the insertion positions of neo cassette, the loxP (closed triangle) and FRT (open triangle) sites, and the pgk-TK expression cassette are indicated. The BamHI restriction digestion sites, the position of the 5′-probe for Southern blotting, the expected location and size for both the Southern blot products and the genotyping products are also indicated. (B) Southern blot results of BamHI digested DNA from *Grp94* WT (+/+) and heterozygous (+/−) siblings. The size of each band is indicated. (C) Genotyping results of DNA from *Grp94*+/+ and +/− mice. The sizes of the PCR products are indicated. (D) Genotyping results of DNA from *Grp94*F/+, +/+ and F/F mice. The sizes of the PCR products are indicated.

To achieve this, the targeting vector for *Grp94* was created by inserting a neo cassette flanked by a pair of loxP sequences into intron 1, and a third loxP into intron 2. The neo cassette was inserted in a reverse orientation with respect to the *Grp94* allele. Upon homologous recombination by expression of cre recombinase, exon 2 was deleted (Figure 1A). To avoid the complication of removing the neo cassette, a pair of FRT sequences was also added adjacent to the loxP sites, flanking the neo cassette in a tandem manner [39]. Removal of the neo cassette by expressing β-actin-driven flp recombinase generated a floxed allele containing two loxP sites flanking exon 2 (Figure 1A). Subsequent removal of exon 2 by cre recombinase generated the knockout (KO) allele (Figure 1A).

The targeting vector was electroporated into ES cells and subjected to G418 and ganciclovir selection. Two out of 600 positive clones were found to contain homologous recombination into the wild-type (WT) allele. One of the clones was able to generate two viable chimeric founder mice, as confirmed by both genotyping and Southern blotting. The chimeric founder mice were mated with WT female C57BL/6J mice and F1 mice with germline transmission of the loxP-FRT targeted allele were generated. To generate the *Grp94* conventional KO allele, the loxP-FRT *Grp94* mice were first mated with an EIIA-cre transgenic mouse. The chimeric progeny with the recombination of the first and third loxP were mated with WT female C57BL/6J mice to generate the pure KO allele, and to segregate the KO allele from the EIIA-cre transgene. The genotype of the *Grp94*+/− mice was validated by Southern blot of tail DNA, using a DNA probe outside the targeting vector (Figure 1A). As expected, after BamH1 digestion, the WT mice yielded a single band of 7 kilobase pairs (kb), and the *Grp94*+/− mice yielded two bands (7 kb and 6 kb) of equal intensity (Figure 1B). The genotype was further confirmed by PCR with specific primer sets designed around exon 1, 2 and 3 to differentiate between the WT allele (336 bp) and the KO allele (593 bp) (Figure 1C). To generate the *Grp94* floxed allele, the loxP-FRT *Grp94* mice were mated with β-actin-driven flp transgenic mouse. The genotype was confirmed by PCR with specific primer sets designed to flank part of intron 1 to differentiate between the WT allele (336 bp) and the floxed allele (420 bp) (Figure 1D).

### *Grp94* Heterozygosity Reduces GRP94 Level but Does Not Alter Basal ER Chaperone Level or Response to ER Stress

**Figure 2:**
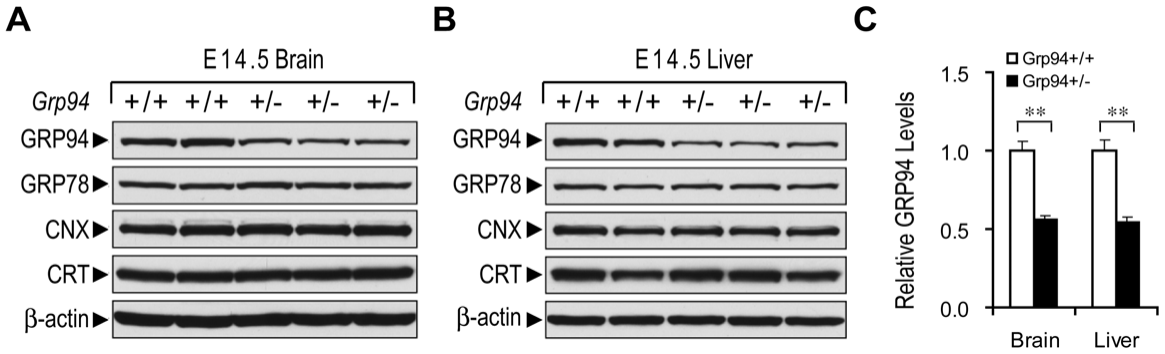
Comparison of ER chaperone expression levels in *Grp94*+/+ and +/− mouse embryonic tissues. (A) Lysates from brains of E14.5 *Grp94*+/+ and +/− embryos were subjected to Western blot with anti-KDEL (to detect GRP94 and GRP78), anti-calnexin (CNX), anti-calreticulin (CRT), and anti-β-actin antibodies. (B) Same as in (A) with cell lysates from livers of E14.5 embryos. (C) Quantitation of GRP94 Western blot results in panel (A) and (B). The quantitation was performed using Quantity One software with the level of GRP94 in *Grp94*+/+ embryonic brain and liver setting as 1. The data are presented as mean±s.e.m. n = 2∼3 per group. These 5 embryos (2 +/+ and 3 +/−) were from one litter. **p≤0.01 (Student's t-test).

Mice bearing the *Grp94*+/+ and +/− genotypes were generated and subjected to analysis. The *Grp94*+/− mice are phenotypically normal and grow at the same rate as WT siblings (data not shown). The *Grp94+/−* mouse embryos are viable and in Western blot analysis, GRP94 protein level in both the brain and liver of the E14.5 embryos was about 50% compared to WT embryos (Figure 2A, B, C). These results are consistent with both the inactivation of one allele of *Grp94* in the heterozygotes and the absence of feedback mechanisms in these embryonic organs to upregulate the remaining WT *Grp94* allele to compensate for the partial loss of GRP94. Previous studies showed that a similar level of reduction of GRP78 in the *Grp78*+/− cells led to a 1.7- and 2-fold increase in the basal level of GRP94 and PDI respectively, but with no change in calnexin (CNX) and calreticulin (CRT) levels [33]. In examining whether there is reciprocal upregulation of ER chaperones in *Grp94* heterozygotes, we observed no change in basal level of GRP78, CNX or CRT, suggesting that in contrast to GRP78, partial GRP94 reduction in mouse embryonic organs does not elicit compensatory increase in basal ER chaperone levels (Figure 2A, B).

**Figure 3:**
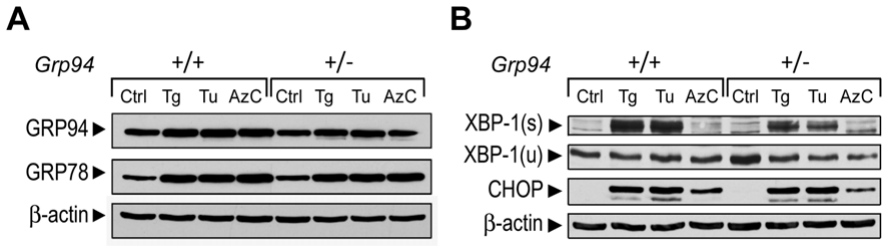
Comparison of ER chaperone expression levels, and UPR signaling in *Grp94*+/+ and +/− mouse embryonic fibroblasts (MEFs). (A) Whole cell lysates from *Grp94*+/+ and +/− MEFs were either untreated (Ctrl), or treated with 300 nM thapsigargin (Tg), 1.5 µg/ml tunicamycin (Tu), or 5 mM azetidine (AzC) for 16 hr and subjected to Western blot with anti-KDEL and anti-β-actin antibodies. (B) Same as in (A) with Western blot with anti-XBP-1, anti-CHOP, and anti-β-actin antibodies. XBP-1(s) and XBP-1(u) indicate the spliced and non-spliced form of XBP-1 respectively.

To test whether cells expressing GRP94 at 50% of the WT level altered their response to ER stress, MEFs isolated from E13.5 *Grp94*+/+ and*+/−* embryos were subjected to various ER stress conditions. We observed that GRP78 induction by all three ER stress inducers, thapsigargin (Tg), tunicamycin (Tu) and azetidine (AzC), was not affected in *Grp94+/−* MEFs (Figure 3A). Further, *Grp94+/−* MEFs did not spontaneously trigger the UPR signaling pathways such as XBP-1 splicing and CHOP induction (Figure 3B). Upon treatment with Tg and Tu, both WT and heterozygous MEFs showed XBP-1 splicing and CHOP induction. Interestingly, in AzC treated cells, the spliced form of XBP-1 [XBP-1(s)] was not evident in either *Grp94*+/+ or +/− MEFs and CHOP induction was milder compared to Tg and Tu treated cells.

### Homozygous *Grp94* Knockout Results in Embryonic Lethality and Growth Factor Dependency of ESCs

**Figure 4:**
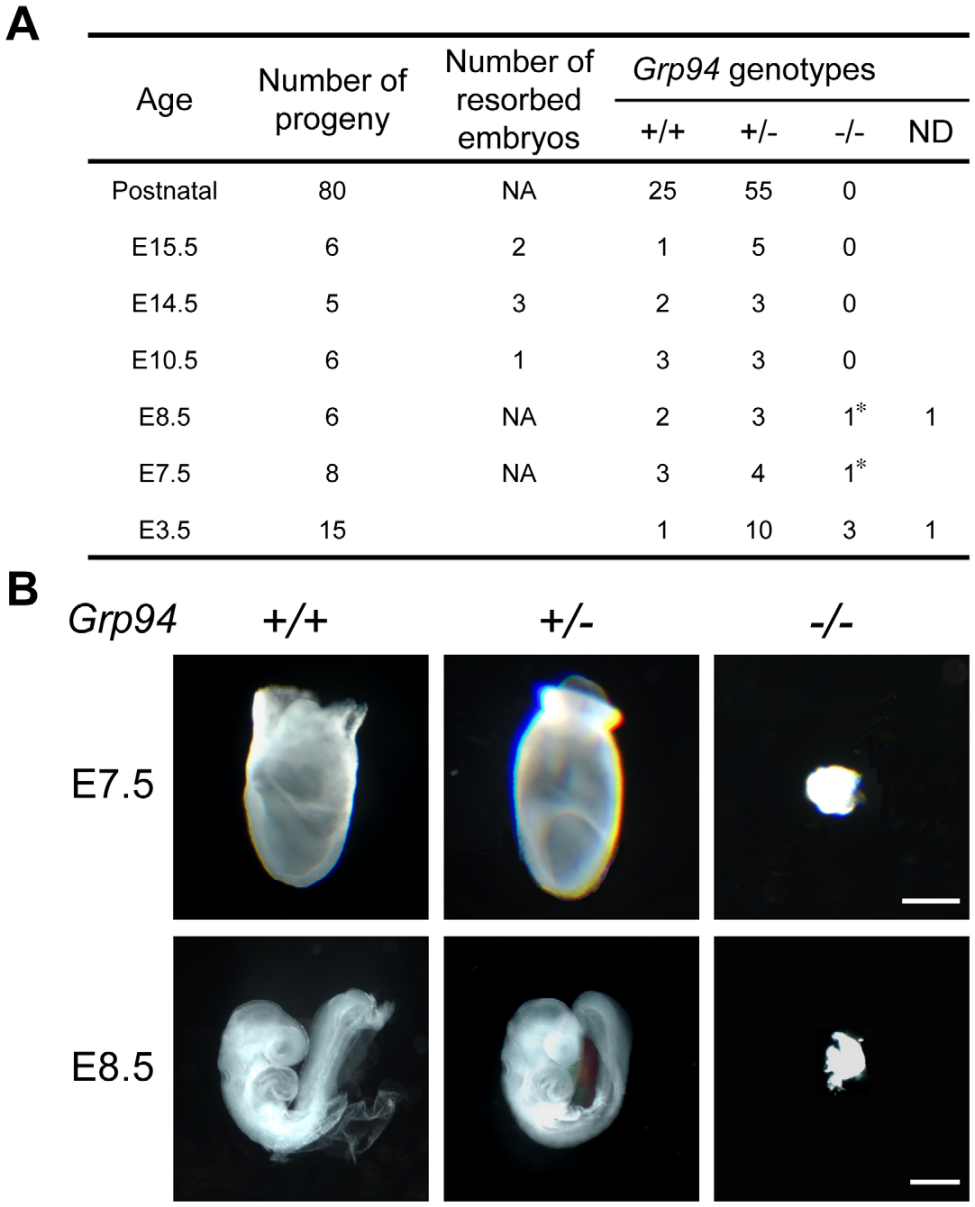
*Grp94* deficiency results in embryonic lethality. (A) Summary of genotypes of embryos at different developmental stages and postnatal progenies from interbred *Grp94*+/− mice. ND, could not be determined by nested PCR. NA, not applicable. (*) denotes deformed embryos. (B) Morphologies of *Grp94*+/+, +/−, and −/− embryos at E7.5 and E8.5. Embryos were genotyped through PCR. Scale bars represent 200 µm (upper panel), 300 µm (lower panel).

**Figure 5:**
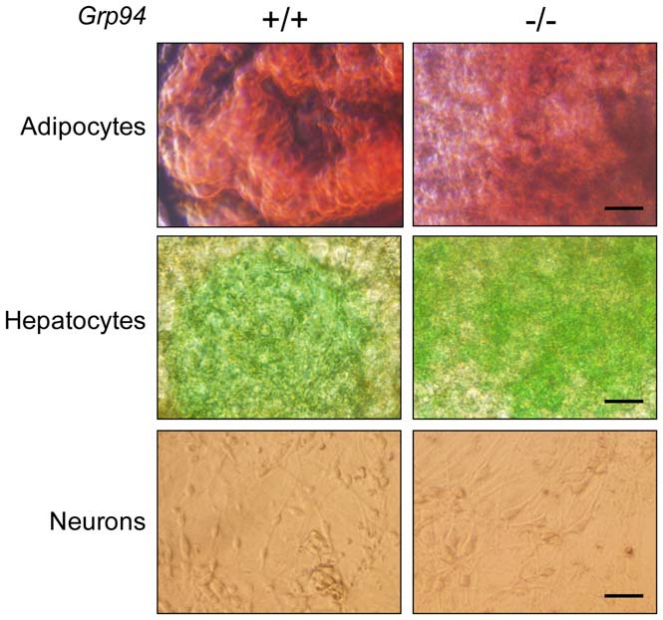
Differentiation properties of *Grp94*+/+ and −/− embryonic stem cells (ESCs). *Grp94*+/+ and −/− ESCs were able to differentiate into adipocytes (top panel), hepatocytes (middle panel) and neurons (bottom panel). The adipocytes and hepatocytes were indicated by oil red and indocyanine green staining positive cells, respectively. The neurons were recognized by their morphology. Scale bars represent 200 µm.

When the *Grp94+/−* mice were intercrossed, out of the 80 pups born, the ratio of +/+, +/− and −/− genotype was 25∶55∶0 respectively (Figure 4A). This suggests that *Grp94−/−* embryos were not viable, whereas *Grp94+/−* mice were born in the expected Mendelian ratio. Further analysis showed that the *Grp94−/−* embryos were detectable by nested PCR at E3.5 up to E8.5. By E10.5 and later, no such genotype of embryos was detected and resorbed embryos were evident. Morphological examination of the embryos revealed that at E7.5 and E8.5, the *Grp94*−/− embryos were much reduced in size with no recognizable structure, compared to normally developing *Grp94*+/+ and +/− embryos (Figure 4B). The survival of the *Grp94*−/− embryos past blastocyst stage afforded the opportunity to establish *Grp94*−/− ESC line for ascertaining GRP94 function *in vitro*. Two different culture mediums were used in our attempts to derive *Grp94*−/− ESC lines. The results were summarized in Table 1. Using 44 blastocysts which generated 35 outgrowths, 18 ESC lines were established when they were cultured in the Knockout (KO) DMEM with 15% knockout serum replacement and 5% fetal bovine serum with standard supplements. Surprisingly, none of the 18 lines contained the *Grp94*−/− genotype. After insulin-transferrin-selenium (ITS) was added to the same medium only a single *Grp94*−/− ESC line was established among 10 ESC lines derived from 24 outgrowths from 30 blastocysts. This implies that exogenous growth factors are uniquely required for *Grp94*−/− ESC establishment. Once the lines were established, we observed that ITS was not required for the maintenance of the *Grp94*−/− ESCs, although upon freezing and thawing, the survival rate of the *Grp94*−/− ESCs was lower compared to wild-type cells. In order to investigate the differentiation ability of the GRP94 null ESCs, paired *Grp94*+/+ and −/− ESCs were utilized. Our results showed that both genotypes could be induced to differentiate into adipocytes (oil red positive cells), hepatocytes (indocyanine green positive cells) and neurons (Figure 5). The results imply that *Grp94*−/− ESCs can differentiate into cells of all three germ layers: ectoderm (neurons), mesoderm (adipocytes) and endoderm (hepatocytes). However, the *Grp94*−/− ESCs are unable to differentiate into cardiomyocyte-like cells in contrast to *Grp94*+/+ ESCs which can form contracting cells similar to cardiomyocytes (Supplementary Video S1).

**Table 1:** Summary of embryonic stem cell (ESC) lines derived from *Grp94+/−* mice interbred.

44 blastocysts 35 outgrowths 0 KO/18 ESC lines	30 blastocysts 24 outgrowths 1 KO/10 ESC lines
KO DMEM	KO DMEM
15% KSR	15% KSR
5% FBS	5% FBS
2 mM L-Glutamine	2 mM L-Glutamine
10 mM NEAA	10 mM NEAA
1 x Penn/Strep	1 x Penn/Strep
0.2 mM 2-ME	0.2 mM 2-ME
1000 U/ml LlF	1000 U/ml LlF
	**1X ITS**

Abbreviations: KO, knockout; DMEM, Dulbecco's modified eagle medium; KSR, knockout serum replacement; FBS, fetal bovine serum; NEAA, non-essential amino acid; 2-ME, β-mercaptoethanol; ITS, insulin-transferrin-selenium; LlF, leukemia inhibitory factor.

### Compensatory Upregulation of Specific ER Chaperones in GRP94 Null ESCs

**Figure 6:**
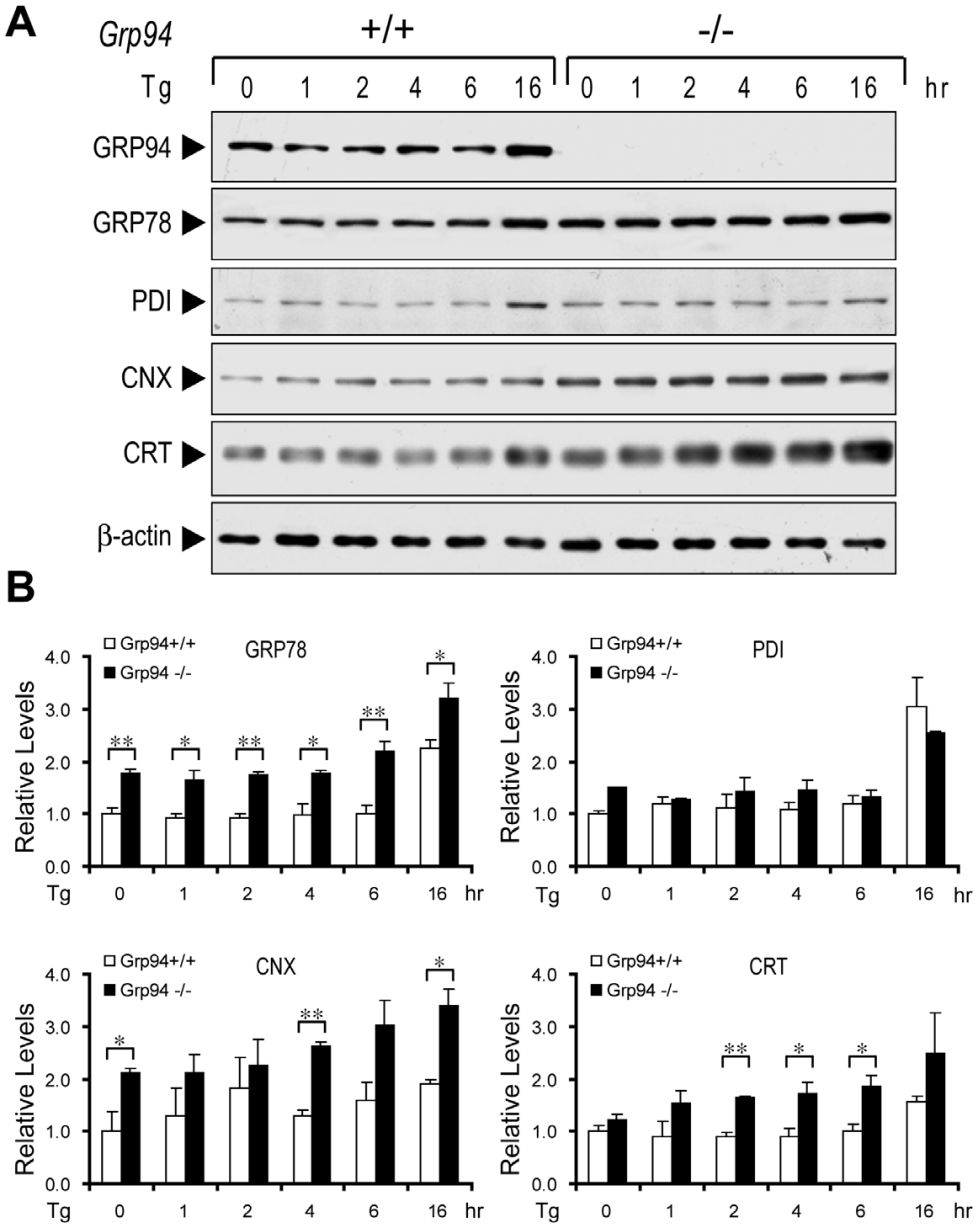
Upregulation of ER chaperones in GRP94 null ESCs. (A) ER chaperones GRP78, CNX and CRT but not PDI were upregulated in *Grp94*−/− ESCs. *Grp94*+/+ and −/− ESCs were treated with 300 nM ER stress inducer thapsigargin (Tg) for the indicated time period (hrs). The whole cell lysates were subjected to Western blot. (B) Summary of the Western blot results of panel (A), which was repeated 2 to 3 times. Quantitation of the Western blots was performed using Quantity One software with the level of each chaperone in *Grp94*+/+ ESCs at 0 hr set as 1. The data are presented as mean±s.e.m. *p≤0.05, **p≤0.01 (Student's t-test).

The consequence of homozygous knockout of GRP94 on basal and ER stress induced chaperone levels was studied utilizing the ESCs. First, we confirmed by Western blot that the *Grp94*−/− cells are devoid of GRP94 (Figure 6A). Interestingly, compensatory upregulation of basal level of GRP78 and CNX was evident in the *Grp94*−/− cells and these increases were further enhanced in Tg stressed cells (Figure 6A, B). For CRT, the increase was also apparent and reached statistical significance upon Tg treatment (Figure 6A, B). Interestingly, for PDI, the increase, if any, was minor; and may even slightly decrease upon 16 hr of Tg treatment in the *Grp94* null cells (Figure 6A, B). These results show that specific elimination of GRP94 triggers a regulatory circuit to enhance expression of specific ER chaperones which include GRP78, CNX and CRT.

### Suppression of ER-stress Induced XBP-1 Splicing in GRP94 Null ESCs

**Figure 7:**
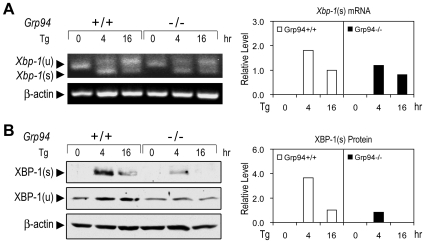
Perturbation of specific UPR targets in GRP94 null ESCs. (A) Effect of GRP94 depletion on *Xbp-1* mRNA splicing. The RNA extracts of *Grp94*+/+ and −/− ESCs treated with Tg for indicated hrs were subjected to RT-PCR. The positions of the PCR products of both the unspliced (u) and spliced (s) *Xbp-1* are indicated by arrows. The *Xbp-1*(s) mRNA level was quantitated using Quantity One software and was normalized against β-actin. The level of *Xbp-1*(s) in *Grp94*+/+ ESCs treated with Tg for 16 hr was set as 1. (B) Reduction of the spliced form of XBP-1 protein in GRP94 null ESCs. Whole cell lysates of *Grp94*+/+ and −/− ESCs treated as in panel (A) were subjected to Western blot. The protein level was quantitated and normalized against β-actin. The level of XBP-1(s) in *Grp94*+/+ ESCs treated with Tg for 16 hr was set as 1. These experiments were repeated three times.

Since GRP78 and GRP94 are components of chaperone complex and GRP78 is known to regulate UPR signaling through interaction and inhibition of transmembrane ER signaling molecules, we investigated whether complete depletion of GRP94 will affect these pathways. *Grp94*+/+ and −/− ESCs were subjected to Tg treatment and the induction of UPR targets were examined. XBP-1 splicing is a major downstream target of the IRE1 signaling. In *Grp94*+/+ ESCs, Tg treatment for 4 hr induced a high level of *Xbp-1* mRNA splicing, which persisted but at a lower level at 16 hr (Figure 7A). For *Grp94−/−* ESCs, Tg-induced *Xbp-1* mRNA splicing was also observed, however, quantitation of the spliced products showed that at the 4 hr time point, the level of spliced *Xbp-1* mRNA was reduced by about 30% compared with *Grp94+/+* ESCs. At the XBP-1 protein level, compared to the *Grp94*+/+ ESCs which showed robust induction of XBP-1(s) at 4 hr and residual amount still present at 16 hr, *Grp94−/−* ESCs showed a 75% reduction of XBP-1(s) at 4 hr and non-detectable amount at 16 hr (Figure 7B). The protein level of the unspliced form of XBP-1 [XBP-1(u)] was relatively constant throughout the Tg treatment period for the *Grp94−/−* ESCs, compared to a slight elevation in the Tg treated *Grp94*+/+ ESCs (Figure 7B).

**Figure 8:**
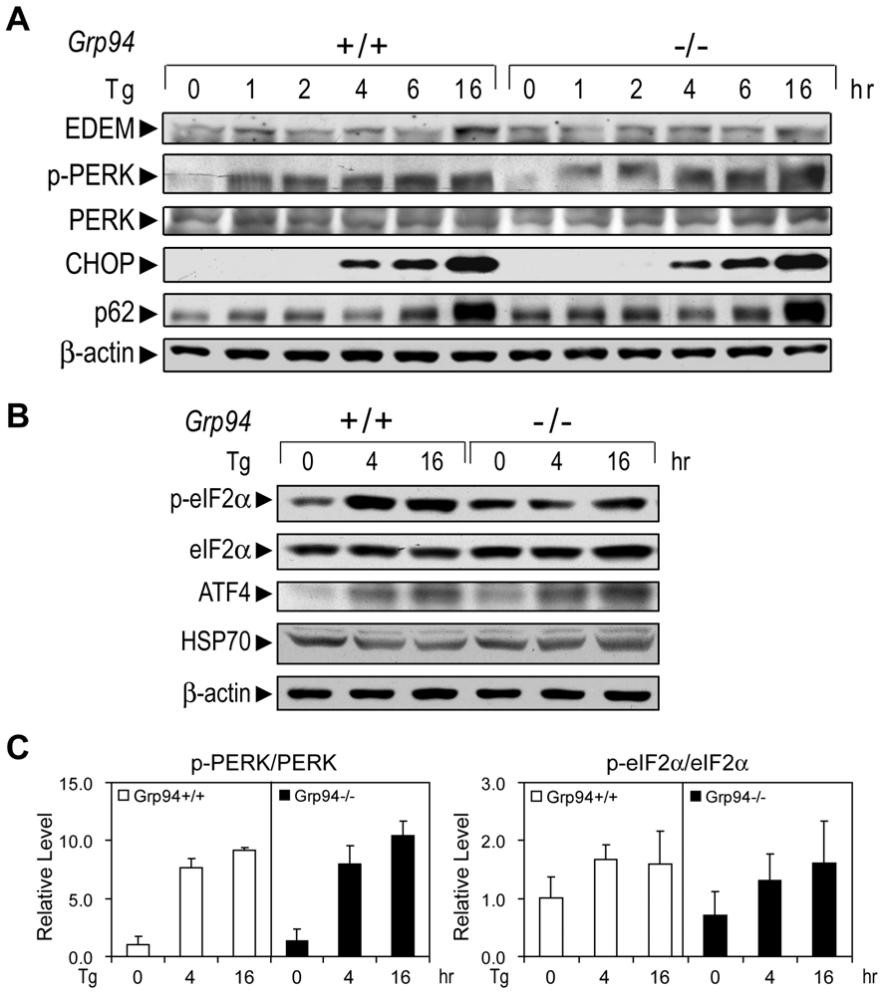
GRP94 deficiency has no effect on some UPR targets. (A) Representative Western blot results on the effect of GRP94 depletion on UPR targets. *Grp94*+/+ and −/− ESCs were treated with 300 nM Tg for the indicated time (hr). The whole cell lysates were subjected to Western blot to detect the level of EDEM, PERK phosphorylation, total PERK, CHOP and p62. (B) The same as in (A) with Tg treatment for 0, 4 and 16 hr. The level of eIF2α phosphorylation, total eIF2α, ATF4 and HSP70 were measured by Western blot. These experiments were repeated two to four times. (C) The levels of p-PERK and p-eIF2α were quantitated and normalized against total PERK and eIF2α, respectively. The data are presented as mean±s.e.m. The experiments were repeated two to four times.

As a downstream target of XBP-1, Tg-induction of EDEM was also reduced in *Grp94*−/− ESCs (Figure 8A). In contrast, other UPR targets and cellular proteins were only mildly affected or completely unaffected by GRP94 depletion. For example, Tg-induced phosphorylation of PERK was only slightly increased in *Grp94*−/− ESCs at 16 hr, with no corresponding increase of CHOP (Figure 8A). The level of p62, a cytosolic protein which binds to polyubiquitinated proteins and targets them to the autophagy machinery for degradation [40] was also not affected in the Tg-treated cells of both genotypes (Figure 8A). There were substantial variations on the Tg-induced phosphorylation of eIF2α in the *Grp94*−/− ESCs however on the average there was no increase (Figure 8B and data not shown). Tg-induction of ATF4 was mildly increased while the level of HSP70, a major heat shock protein related to GRP78, was not affected (Figure 8B). Quantitation of p-PERK and p-eIF2α levels from several independent experiments showed no statistically significant change between the two genotypes (Figure 8C). Thus, the minor differences observed are likely due to experimental variations in the cultured ESC system.

**Figure 9:**
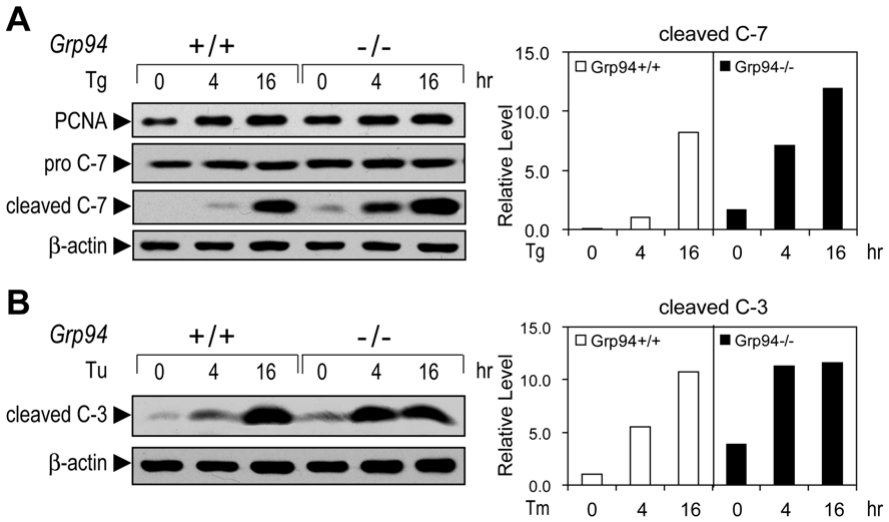
GRP94 protects ESCs from ER stress-induced cell death. (A) Representative Western blot results on effect of GRP94 depletion on cell proliferation and apoptosis. *Grp94*+/+ and −/− ESCs were treated with 300 nM Tg for the indicated time (hr). The whole cell lysates were subjected to Western blot with antibodies against PCNA, caspase-7 (C-7) and β-actin. Both pro C-7 (the uncleaved form) and cleaved C-7 (active form) were detected by the anti-C-7 antibody. The cleaved C-7 protein level was quantitated and normalized against β-actin. The level of cleaved C-7 in *Grp94*+/+ ESCs treated with Tg for 4 hr was set as 1. (B) The same as in (A) with 1.5 µg/ml Tunicamycin (Tu) treated cells. The Western blots were performed with antibodies against cleaved caspase-3 (C-3) and β-actin. The cleaved C-3 protein level was quantitated and normalized against β-actin. The level of cleaved C-3 in *Grp94*+/+ ESCs without Tu treatment was set as 1. These experiments were repeated two times.

Upon Tg treatment, *Grp94+/+* and −/− ESCs showed similar levels of proliferating cell nuclear antigen (PCNA), suggesting no major alteration in the proliferative properties (Figure 9A). However, there was a substantial increase in the level of cleaved caspase-7 (C-7), implying that GRP94 depletion in the *Grp94−/−* ESCs sensitizes the cells to Tg-induced apoptosis (Figure 9A). This result was further confirmed by caspase-3 (C-3) activation. Consistent with C-7, the level of cleaved C-3 was also elevated in *Grp94*−/− ESCs, following treatment with ER stress-inducer Tu (Figure 9B).

### Differential Expression of GRP94 in Adult Mouse Tissues

**Figure 10:**
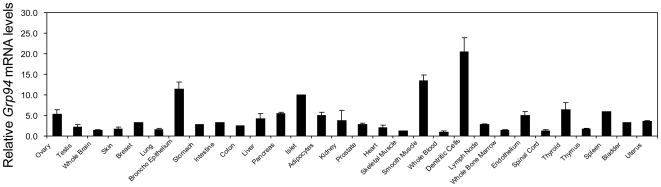
Relative *Grp94* mRNA levels in adult mouse tissues and primary cells. The data were from Amazonia microarray database. All the data are presented as mean±s.e.m, with the level of *Grp94* mRNA in whole blood set as 1.

**Figure 11:**
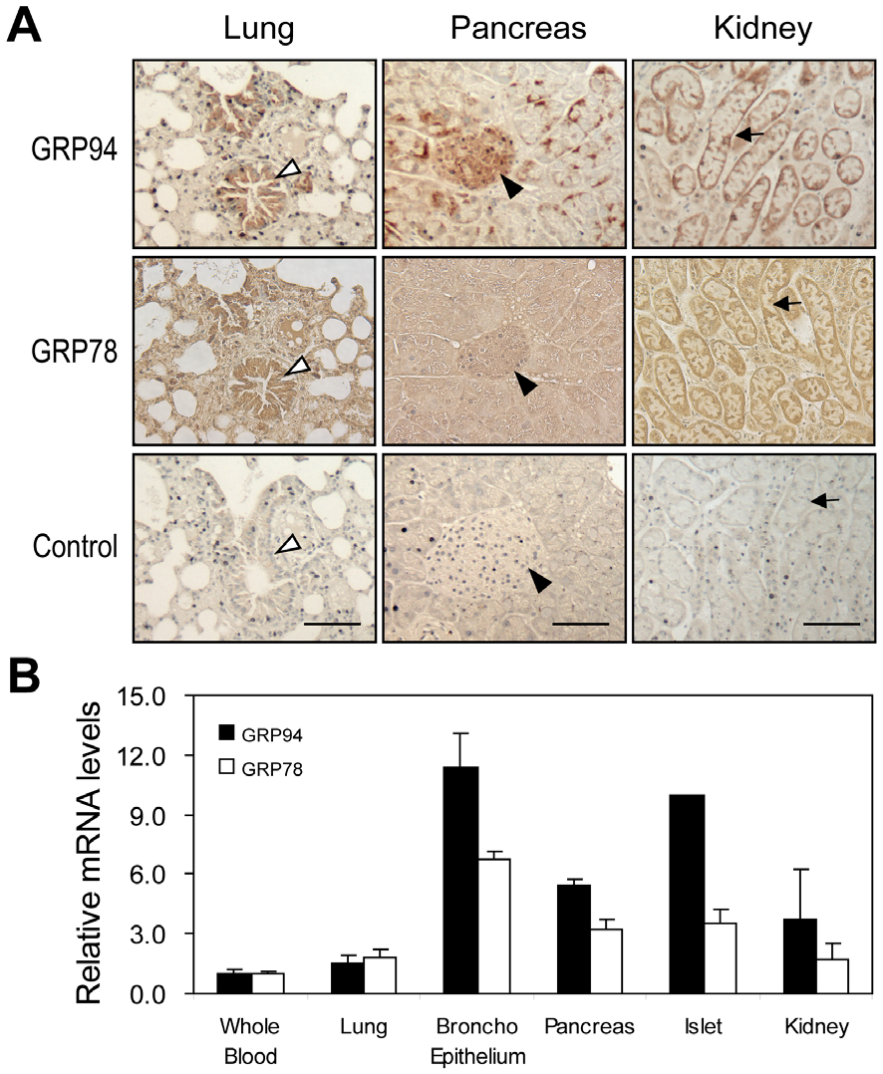
GRP94 and GRP78 staining and mRNA expression in adult mouse tissues. (A) Immunohistochemical staining for GRP94 and GRP78 in adult mouse lung, pancreas and kidney tissues. The GRP78 staining and control staining (without primary antibody) were presented to show the specificity of the GRP94 antibody. The sections were counterstained with hematoxylin. Brown color depicts GRP94 and GRP78 staining. White arrowheads indicate lung bronchial epithelium. Black arrowheads indicate pancreatic islet. Black arrows indicate renal cuboidal epithelium. Scale bars represent 50 µm. (B) Relative levels of *Grp94* and *Grp78* mRNA in adult mouse lung, broncho epithelium, pancreas, islet and kidney, with the level of *Grp94* and *Grp78* mRNA in whole blood set as 1.

Analysis of a microarray database (Amazonia) revealed that *Grp94* mRNA is expressed at relatively low levels in most mouse adult tissues (Figure 10). Strikingly, the level is about 20-fold higher in dendritic cells, 12-fold higher in both smooth muscle and lung bronchial epithelium and 10-fold higher in pancreatic islet, with the *Grp94* mRNA level in whole blood setting as 1. To correlate these mRNA data with protein expression level, we examined the level of GRP94 in mouse adult organs such as lung, pancreas and kidney by immunohistochemistry. The results showed that while GRP94 protein was expressed at low basal level in the lung, pancreas and kidney, it is strikingly high in the lung bronchial epithelium, pancreatic islets of Langerhans and kidney cuboidal epithelium (Figure 11). For comparison, GRP78 staining was elevated in the lung bronchial epithelium and kidney cuboidal epithelium, however, it was not as prominent as GRP94 in the pancreatic islets of Langerhans (Figure 11A). The immunohistochemical staining results for GRP94 and GRP78 were consistent with relative levels of *Grp94* and *Grp78* mRNA from the microarray database in the specific tissues being examined (Figure 11B). Thus, high level expression of GRP94 in specific cell types of the lung, pancreas and kidney suggests that GRP94 could play an important role in homeostasis in these cell types, which may represent excellent targets for conditional knockout of GRP94 in mouse models for further probing of GRP94 function *in vivo*.

## Discussion

Endoplasmic reticulum stress has emerged to play important roles in mammalian development and human diseases. In order to better understand the molecular pathways that allow cells to adapt to ER homeostasis, we focus on ER chaperones which are critical not only for quality control of proteins processed in the ER, but may also directly or indirectly regulate ER signaling in response to ER stress and maintenance of calcium homeostasis [19]. GRP94 is highly conserved in vertebrates but not in yeast and has been well documented to be inducible by a variety of metabolic stresses, suggesting that it is uniquely required for functions in vertebrates not shared by yeast. As documented in earlier reports [28], [34], [41], creation of model organisms with targeted mutation of GRP94 allows comprehensive analysis of its *in vivo* function.

Based on our finding that knockout of GRP78, another major ER chaperone closely associated with GRP94 in function and regulation, results in embryonic lethality around peri-implantation (E3.5) stage [33], mice deficient in GRP94 may also die during embryonic development. Hence, using the cre-loxP system, we designed a mutated allele of *Grp94* that allows both the traditional knockout and conditional knockout in a tissue or time specific manner. Since exon 1 of *Grp94* only contains 49 bp, the positioning of the neo cassette into intron 1 coupled with expected frame shift ensures early disruption of the GRP94 of the coding sequence and elimination of GRP94 with any functional domain. Utilizing heterozygous and homozygous knockout *Grp94* cells and mice derived from this strategy, we examined how complete and partial GRP94 depletion altered development and the ER stress response. We discovered that deficiency of GRP94 is distinct in many different aspects from deficiency in GRP78, which has an established role in regulating ER signaling in addition to being a major chaperone.

While both heterozygous *Grp78* and *Grp94* mice show no obvious abnormal phenotype, cells with partial GRP78 knockdown upregulate GRP94 and PDI; however no such compensation is observed for partial GRP94 deficiency. Nonetheless, in cells completely devoid of GRP94, we discovered that not only is GRP78 upregulated, but also CNX and CRT. Interestingly, PDI appears to be substantially less affected or unaffected. Thus, it is possible that feedback mechanisms exist to compensate the complete loss of GRP94 by increasing GRP78, a partner protein of GRP94 in the GRP78/GRP94 chaperone system, and also of the CNX/CRT chaperone system, but apparently not the thiol oxidoreductases such as PDI. Collectively, these results imply that cells have abilities to adjust the ER chaperone balance in response to depletion of key chaperones such as GRP94 and GRP78.

During the course of creation of our mouse models and analysis of the phenotypes, it has been reported that homozygous knockout of *Grp94* through insertion of a *neo* resistance cassette into the coding region at the end of exon 3 (61 amino acids into the mature protein) of the murine *Grp94* gene leads to embryonic lethality around day 7 of gestation [34]. The embryos failed to develop mesoderm, primitive streak, or proaminotic cavity, and GRP94 is essential for mesoderm induction and muscle development due to deficiency in IGF-II secretion. Our *Grp94* knockout mouse model targets exon 2 of the *Grp94* allele. Additionally, our model differs from the previous *Grp94* KO model [34] in that in our model the *neo* resistance cassette was inserted into intron 1, and exon 2 was completely eliminated through flox recombination. While we also observed embryonic lethality around E7, the ratio of pups born for the +/+, +/− and −/− genotype was 25∶55∶0 for our model eliminating exon 2, whereas the model disrupting exon 3 [34] reported a ratio of 414∶515∶0. Since the *Grp94+/−* pups in our model were born in the expected Mendelian ratio of 1∶2 with respect to *Grp94+/+* pups, our results suggest that the *Grp94+/−* embryos in our model were fully viable. This is consistent with our observation that the growth and biochemical properties of *Grp94+/+* and *+/−* ESCs and MEFs isolated from our *Grp94* mouse model are indistinguishable (data not shown). The floxed *Grp94* allele that we created is capable of achieving conditional knockout of the *Grp94* gene, as *Gp94*F/-; Purkinje cell-cre mice have been recently created to deplete GRP94 completely from Purkinje cells, showing that GRP94 is not required for the survival of Purkinje cells [42]. Our conditional deletion model created here is distinct from another conditional deletion model of *Grp94* which targets exon 1, which has been used to achieve specific deletion of *Grp94* in macrophages and determining its function in innate immunity and macrophage homeostasis [28].

Our difficulty in establishing *Grp94*−/− ESC lines from E3.5 blastocysts in routinely used ESC culture medium suggests that some critical component is missing or not of sufficient quantity. The recent discovery that *Grp94* deficiency blocks IGFII secretion [34], [35] and our observation that addition of ITS supplement yielded successful ESC line derivation supports the stringent requirement of exogenous growth factors for *Grp94* null ESC establishment. Another contributing factor may be the genetic background of the *Grp94* blastocysts as the mice used in our study have been backcrossed to the C57BL/6 background, and establishment of C57BL/6 mouse ESCs has been reported to be more difficult [43]. Finally, with the GRP94 null ESCs independently derived from our *Grp94* KO mice, we directly confirmed the requirement of GRP94 for ESC differentiation into cardiac muscle cells.

The ESC lines with complete elimination of GRP94 also provide us with a unique opportunity to study whether GRP94, as a partner protein of GRP78, contributes to ER stress signaling. In the case of GRP78 knockdown or knockout, UPR signaling targets such as p-eIF2α, CHOP and GADD34 are activated in the absence of stress, clearly showing GRP78 suppresses their activation under non-stress conditions [42], [44]. Here we report the novel observation that the XBP-1(s) protein level was substantially reduced following Tg treatment in *Grp94* null ESCs compared to wild-type control. Interestingly, for both *Grp94*+/+ and −/− ESCs, PERK phosphorylation persisted up to 16 hr after Tg treatment, whereas in most other cell lines, ER stress-induced PERK phosphorylation was transient and subsided within a few hours of ER stress. On the other hand, in contrast to GRP78 depletion, no spontaneous induction of CHOP is observed in cells depleted of GRP94 and the kinetics of its induction by Tg is unaffected. This suggests that while GRP94 may act in concert with GRP78 in folding protein intermediates, its depletion results in de-regulation of the specific UPR targets quite distinct from those affected by GRP78 deficiency.

How might GRP94 depletion suppresses ER-stress induced XBP-1 splicing? Both GRP94 and XBP-1 have been implicated in immune function [28], [45]–[47]. Using mouse models of specific knockout of *Grp94* in macrophages, it was demonstrated that GRP94 is the master chaperone for Toll-like receptors (TLR) and is important in the innate function of macrophages [28]. Analysis of B-cell specific GRP94 null mice further revealed that GRP94 optimizes B-cell function serving as chaperones for integrin and TLR but not immunoglobulins [29]. Recently, it was discovered that TLR4 and TLR2 specifically activated the UPR sensor IRE1α and its downstream target XBP-1 in macrophages [48]. Since GRP94 is required for maturation of TLRs, including TLR4 and TLR2, it may regulate XBP-1 activation through the TLRs. Future studies will be required to test this and other mechanisms. The differential effect of GRP94 depletion on XBP-1 splicing but not other UPR signaling pathways also supports the notion that UPR sensors can be dissociated and selectively regulated [49].

Through analysis of a microarray database, we discovered that GRP94 expression is strikingly high in dendritic cells, smooth muscle and lung bronchial epithelium. The microarray data, which is supported by tissue staining results, predicts potential role of GRP94 in immunity and function/survival of specific cell types in the lung, pancreas and kidney. Future studies with conditional KO of *Grp94* in these cell types will address these important issues. Furthermore, the role of GRP94 is human diseases such as cancer, diabetes and neurodegeneration can be also achieved through the use of the mutant mouse models.

## Materials and Methods

### Generation of the loxP-FRT *Grp94* Targeting Vector

An 8 kb DNA fragment for *Grp94* gene (spanning from the promoter region to intron 5) was isolated from a 129/Sv-derived lambda FixII mouse genomic library (a gift from Dr. Robert Maxson, University of Southern California (USC) Keck School of Medicine). Using this as a template, subcloning and PCR reactions were performed and fragments were subcloned into the pBluescript KS vector to create the *Grp94* loxP-FRT targeting vector. This vector was constructed by inserting a neo cassette, flanked by a pair of loxP sites and a pair of FRT sites (kindly provided by Dr. Hualin Fu, USC), into a Stu I site in intron 1, a third loxP site into intron 2 and a pgk-TK (thymidine kinase) expression cassette at the 3′ end of the construct as a negative selection marker (Figure 1A). The targeting vector was linearized by NotI and was purified and electroporated into 129/Sv-derived embryonic stem cells (ESCs). The ESCs were selected by G418 and ganciclovir, and resistant colonies were screened through PCR and Southern blotting for homologous recombination.

### Generation of *Grp94*+/− and F/F Mice

All animals were housed under pathogen-free conditions and experiments were performed in accordance with the USDA Animal Welfare Regulations on Humane Care and Use of Laboratory Animals. All animal experimental protocols were approved by the University of Southern California Institutional Animal Care and Use Committee. The selected ESC clone was subjected to subcloning to achieve a higher purity of targeted ESCs and injected to blastocysts from the C57BL/6J female mouse. Chimeric founder mice were genotyped by PCR. The founder mouse with the correct genotype was mated with C57BL/6J female mice to generate the *Grp94* loxP-FRT mice. The progenies were subjected to PCR genotyping. The primers for identifying loxP-FRT allele were FL-F: 5′-CTCCTGAGACCGAAAAGGACT-3′ and FL-R: 5′-AGGATTGGGAAGACAATAG CAG-3′, with a 440 base pair (bp) product. To generate the *Grp94* conventional knockout (KO) (−) allele, the loxP-FRT *Grp94* mice were mated with an EIIA-Cre transgenic mouse (Jackson Laboratory) and progenies were genotyped by PCR. To generate the *Grp94* floxed (F) allele, the loxP-FRT mice were mated with β-actin-driven flp transgenic mice (Jackson Laboratory) and PCR was used to confirm the successful generation of the F allele. The primers used to differentiate WT, KO and F alleles were 94KOniL: 5′-GCTGTGTCCTGCTGACCTTCG-3′, 94KOniR: 5′-TACCTCACCGATTGAAAAGC-3′ and PAS3: 5′-TGATCAGCGATCGCCAAAAGTCCTTAGGGAGG-3′. 94KOniL and PAS3 were used to detect both the WT allele with a product of 336 bp and the F allele with a product of 420 bp. 94KOniL and 94KOniR were used to detect the KO allele with a product of 593 bp. The *Grp94*+/− progeny bearing a KO allele was mated to C57BL/6J female mice to segregate the KO allele from the EIIA-Cre transgene. The *Grp94*+/− mice used in this study were backcrossed to C57BL/6 background for 3 to 4 generations.

### Isolation, Culture and Drug Treatment of MEFs

Mouse embryonic fibroblasts (MEFs) were isolated from E13.5 embryos. Briefly, the embryo was washed twice in PBS after removal of head and internal organs, disaggregated with an 18-gauge needle in 0.25% trypsin-EDTA, and were incubated at 37°C for 2–3 min before adding DMEM with 10% FBS and 1% penicillin and streptomycin. The cells were split after 2 days in culture and MEFs were used for experiments. For drug treatment, the cells were treated with 300 nM thapsigargin (Tg), 1.5 µg/ml tunicamycin (Tu), or 5 mM azetidine (AzC) for 16 hr prior to harvest.

### Generation of ESCs and Differentiation Analysis

ESCs were derived from interbred *Grp94*+/− mice (backcrossed to C57BL/6 background for 3 or 4 generations). The blastocysts from E3.5 pregnant female mice were recovered and cultured on MEFs. One *Grp94*−/− and multiple +/+ and +/− ESC lines were established. *Grp94*+/+ and −/− ESCs were used to evaluate the role of GRP94 in ESC differentiation potential. The detailed protocol for ESC differentiation has been described previously [34]. In brief, both lines were differentiated via embryoid body (EB) formation and treatment with or without retinoic acid. In two independent experiments, EBs were generated from both lines. Oil red and indocyanine green were used to identify adipocytes and hepatocyte-like cells, respectively. Neuronal cells were identified by the morphology. No beating foci were observed in EBs derived from *Grp94*−/− ESCs while the EBs derived from *Grp94*+/+ ESCs produced many such foci.

### Southern Blotting

The BamHI digested DNA (from tail biopsy or ESCs) was run on a 1% agarose gel at 12 volt overnight. The DNA was then transferred to a nylon membrane for Southern blotting using the 5′ external probe described above for ESCs screening.

### Western Blotting

Brains and livers dissected from E14.5 mouse embryos were homogenized in RIPA buffer with a Dounce homogenizer, followed by centrifugation at 13,000 g at 4°C for 15 min. MEFs and ESCs were also lysed in RIPA buffer followed by centrifugation (13000 g, 151 min) after 3 freeze-thaw cycles. The Western blots were performed as previously described [50]. The primary antibodies used included the following: mouse anti-KDEL(10C3) (1∶1000), rat anti-GRP94 (1∶2500), rabbit anti-calnexin (1∶2000), and rabbit anti-calreticulin (1∶2000) from Stressgen; goat anti-GRP78(C20) (1∶5000), rabbit anti-PERK (H-300) (1∶500), rabbit anti-ATF4 (CREB-2) (1∶1000), goat anti-HSP70 (K-20) (1∶1000), mouse anti-PCNA (PC10) (1∶1000), mouse anti-CHOP (1∶1000), goat anti-EDEM (1∶500) and rabbit anti-XBP1 (1∶500) from Santa Cruz Biotechnology; rabbit anti-phospho-PERK (Thr980) (1∶500), mouse anti-cleaved-caspase-7 (Asp198), rabbit anti-phospho-eIF2α (1∶1000), rabbit anti- eIF2α (1∶1000) and rabbit anti-cleaved caspase-3 (Asp175) (1∶500) from Cell Signaling; rabbit anti-PDI (1∶2000) from Assay Designs; mouse anti-β-actin (1∶5000) from Sigma-Aldrich; and rabbit anti-p62 (SQSTM1) (1∶1000) from BIOMOL. The experiments were repeated 2 to 5 times. Protein levels were visualized by Western blot films and then quantitated using Quantity One software. Phospho-PERK was quantitated against total PERK, and phospho-eIF2α was quantitated against total eIF2α. Both spliced and unspliced XBP-1 were quantitated against β-actin. All the other proteins were quantitated against β-actin.

### RT-PCR Analysis of *Xbp-1* mRNA Splicing

The cells were either untreated or treated with 300 nM ER stress inducer thapsigargin (Tg) for 4 hr or 16 hr. After that, total RNA was extracted using TRI reagent (Sigma-Aldrich) following the manufacturer's instructions. First-strand cDNA was synthesized with SuperScript II (Invitrogen). To detect both unspliced and spliced *Xbp-1* mRNA, PCR was performed as previously [51]. The primers for PCR of *Xbp-1* and β-actin were described previously [42]. These experiments were repeated two to three times.

### Immunohistochemical Staining

Mouse tissues were fixed overnight in 10% buffered formalin and embedded in paraffin using standard protocols. Immunohistochemical staining was carried out as described previously [52]. In brief, Vectastain Elite avidin-biotin complex kit (Vector Laboratories) was used for staining. Paraffin sections were incubated with rat anti-GRP94 antibody (1∶1000) (Stressgen), mouse anti-BiP/GRP78 antibody (1∶200) (BD Biosciences) or without primary antibody (control staining) in blocking solution (1.5% serum in PBS) at 4°C overnight after antigen retrieval with retrivagen A (pH 6.0) (BD Biosciences).

### Microarray Analysis

The *Grp94* and *Grp78* mRNA expression level was downloaded from the Amazonia microarray database, which can be accessed freely on the website: http://amazonia.transcriptome.eu/expression.php?geneId=Hs.192374# (*Grp94*) and http://amazonia.transcriptome.eu/expression.php?geneId=Hs.716396 (*Grp78*). The data we presented in Figure 10 and Figure 11B were from studies performed with embryonic and adult mouse normal tissues (U133A) chip for HSP90B1(*Grp94*) and HSPA5 (*Grp78*).

## Supporting Information

Video S1 Differentiation of Grp94 WT ESCs into cardiomyocyte-like cells. *Grp94*+/+ embryonic stem cells (ESCs) were able to differentiate into cardiomyocyte-like cells indicated by the presence of beating foci in embryoid bodies (EBs). However, beating foci were not observed in EBs derived from *Grp94*−/− ESCs.
(4.53 MB MOV)
